# Antithrombotic Effects of Combined PAR (Protease-Activated Receptor)-4 Antagonism and Factor Xa Inhibition

**DOI:** 10.1161/ATVBAHA.120.314960

**Published:** 2020-09-10

**Authors:** Mohammed N. Meah, Jennifer Raftis, Simon J. Wilson, Vidya Perera, Samira M. Garonzik, Bindu Murthy, J. Gerry Everlof, Ronald Aronson, Joseph Luettgen, David E. Newby

**Affiliations:** 1 British Heart Foundation Centre for Cardiovascular Science, University of Edinburgh, United Kingdom (M.N.M., J.R., S.J.W., D.E.N.).; 2 Bristol-Myers Squibb, Princeton Pike Facility, NJ (V.P., S.M.G., B.M., R.A.).; 3 Brisol-Myers Squibb, Lawrenceville Facility, Princeton, NJ (J.G.E., J.L.).

**Keywords:** apixaban, double-blind method, factor Xa inhibitors, PAR-4 antagonism, platelet aggregation, thrombosis

## Abstract

PAR (protease-activated receptor)-4 antagonism has antiplatelet effects under conditions of high shear stress. We aimed to establish whether PAR4 antagonism had additive antithrombotic activity in the presence of factor Xa inhibition in an ex vivo model of acute arterial injury.

**Approach and Results:**

Fifteen healthy volunteers (29±6 years, 7 women) completed a phase zero double-blind randomized controlled crossover trial. Ex vivo platelet activation, platelet aggregation, and thrombus formation were measured following blood perfusion of low shear and high shear stress chambers. Upstream of the chambers, extracorporeal blood was admixed with (1) vehicle, (2) low-dose apixaban (20 ng/mL), (3) high-dose apixaban (80 ng/mL), (4) BMS-986141 (400 ng/mL), (5) BMS-968141 and low-dose apixaban, or (6) BMS-968141 and high-dose apixaban in 6 sequential studies performed in random order. Compared with vehicle, BMS-986141 demonstrated selective inhibition of PAR4-AP (agonist peptide)–stimulated platelet aggregation, platelet-monocyte aggregates, and P-selectin expression (*P*≤0.01 for all). Total thrombus area was reduced under both low shear and high shear stress conditions for all drug infusions (*P*<0.0001 for all versus vehicle). BMS-968141 reduced total (≤44.4%) and platelet-rich (≤39.3%) thrombus area, whereas apixaban reduced total (≤42.9%) and fibrin-rich (≤31.6%) thrombus area. Combination of BMS-986141 with apixaban caused a further modest reduction in total thrombus area (9.6%–12.4%), especially under conditions of high shear stress (*P*≤0.027).

**Conclusions:**

In the presence of factor Xa inhibition, PAR4 antagonism with BMS-986141 further reduces thrombus formation, especially under conditions of high shear stress. This suggests the potential for additive efficacy of combination PAR4 antagonism and factor Xa inhibition in the prevention of atherothrombotic events.

**Graphic Abstract:**

A graphic abstract is available for this article.

## Abstract 2

Supplemental Digital Content is available in the text.

**Highlights**

- The addition of PAR (protease-activated receptor)-4 antagonism with BMS-986141 to factor Xa inhibition with apixaban reduces total thrombus formation more than apixaban alone under conditions of high shear stress.
- The reduction in total thrombus formation by BMS-986141 is driven by a reduction in high-shear platelet-rich thrombus formation.
- Combination PAR4 antagonism and factor Xa inhibition therapy is a promising potential therapeutic strategy to prevent atherothrombotic events.

PARs (protease-activated receptors) are a group of G-protein–coupled receptors activated by serine proteases. PAR1 and PAR4 are expressed on human platelets, are activated by thrombin,^1^ and act as the primary receptors for thrombin-mediated platelet aggregation.^2^ PAR1 activation causes rapid initial platelet aggregation, whereas PAR4 is activated by higher thrombin concentrations to cause a more prolonged, sustained, and irreversible aggregatory response.^3–6^ This has led to the development of selective PAR4 antagonists. We have previously shown PAR4 antagonism can reduce human platelet-rich thrombus under conditions of high shear stress that are observed within stenosed coronary arteries.^5^ Moreover, preclinical studies in primates have suggested that PAR4 antagonism may be associated with less bleeding than available antiplatelet agents while maintaining antithrombotic efficacy.^7^

Anticoagulant therapies can have additional antiplatelet effects that may impact on overall antithrombotic activity. For example, warfarin inhibits thrombin generation and reduces not only fibrin formation but also PAR1 and PAR4-mediated platelet activation.^8^ Indeed, combinations of antiplatelet and anticoagulant therapies have not always demonstrated additive benefit. For example, in the CARS (Coumarin Aspirin Reinfarction Study), the combination of low-dose warfarin and aspirin therapy was not superior to aspirin therapy alone in the prevention of cardiovascular events.^9^ While meta-analysis suggests higher doses of warfarin (to maintain an international normalized ratio between 2 and 3) in addition to aspirin are effective at reducing cardiovascular events, they are associated with a doubling in major bleeding events.^10^ However, recent clinical trial evidence has demonstrated that combined low-dose factor Xa inhibition and aspirin therapy is more effective than either alone in the prevention of major cardiovascular events in participants with chronic stable disease.^11,12^ This added benefit was at the risk of additional bleeding, and finding the optimal balance between antithrombotic efficacy and bleeding safety remains a challenge for the field. The combination of factor Xa inhibition and an antiplatelet agent with a low bleeding liability would seem attractive.

Factor Xa inhibition reduces thrombin generation by blocking the enzymatic activity of factor Xa and the prothrombinase complex.^13,14^ As such, factor Xa inhibition may interfere with thrombin-mediated PAR1 and PAR4 receptor activation and thereby reduce the potential added efficacy of PAR4 antagonism. We, therefore, wanted to explore how the combination of PAR4 antagonism with factor Xa inhibition would affect thrombus formation. We here assess the efficacy of BMS-986141—a novel PAR4 antagonist—in combination with clinically relevant concentrations of apixaban—a factor Xa inhibitor—in ex vivo model of human thrombus formation.

## Materials and Methods

The data that support the findings of this study are available from the corresponding author upon reasonable request.

### Study Design and Study Population

This was a phase zero double-blind randomized controlled crossover trial. Ex vivo assessments of thrombus formation under conditions of low shear and high shear stress were assessed in an established and validated model of acute arterial injury.^5,15–19^

The study population consisted of men or women between 18 and 45 years of age who had a body mass index between 18 and 35 kg/m^2^ and were willing and able to donate blood. They were fasted for at least 4 hours and verbally confirmed that they had been abstinent from caffeine and alcohol consumption for 24 hours. Participants were excluded if they had a clinically significant illness, were taking regular medication (except oral contraceptives), experienced any recent acute illness or surgery, were pregnant or smokers, or were unable to undergo repeated venepuncture.

### Study Protocol

Participants attended on 2 occasions at the same time of day to undergo 3 sequential and separate chamber studies on each day. They were randomized to 1 of 6 sequences to receive each extracorporeal infusion in a crossover design (Table I in the Data Supplement): (1) study vehicle (99% polyethylene glycol 400/1% Kollidon VA 64) alone or study vehicle plus (2) low-dose apixaban (20 ng/mL), (3) high-dose apixaban (80 ng/mL), (4) BMS-986141 (400 ng/mL), (5) combination low-dose apixaban (20 ng/mL) and BMS-986141 (400 ng/mL), and (6) combination high-dose apixaban (80 ng/mL) and BMS-986141 (400 ng/mL; Figure I in the Data Supplement). These infusions were administered at a rate of 0.2 mL/min into venous blood shortly after leaving the volunteer and before entering the perfusion chamber.

### Study Assessments

#### Ex Vivo Perfusion Chamber

All participants were cannulated with a 17-G cannula in the antecubital fossa. Thrombus formation was assessed using a perfusion chamber.^5,15–19^ In brief, a pump was used to draw blood directly from the antecubital vein through a series of 3 perfusion chambers maintained at 37°C in a water bath. Each chamber contained a strip of porcine aorta that had been stripped of its intimal layer thereby allowing blood to be exposed to conditions and constituents common to arterial media, mimicking deep arterial injury.^20^ Rheological conditions in the first chamber (low shear stress; ≈212 s^−1^) simulate those of patent coronary arteries or high venous flow, whereas those in the second and third chambers (high shear stress; −1690 s^−1^) stimulate stenotic coronary arteries.^21^ Each experimental run lasted exactly 5 minutes with blood flow maintained at a constant rate of 10 mL/min. All studies were performed by the same operator.

#### Blood Sampling and Agonists

All blood samples for pharmacokinetic and platelet function assessments were taken from the effluent of the chamber 2 minutes after commencement of chamber perfusion. PAR4-activating peptide (A-Phe[4-F]-PGWLVKNG) was provided by Bristol-Myers Squibb (Princeton, NJ), ADP by Sigma-Aldrich (Gillingham, United Kingdom), and arachidonic acid by Alpha Laboratories (Eastleigh, United Kingdom). The final agonist concentrations were 200 μM for PAR4-activating peptide, 10 μM for ADP, and 5 mmol/L for arachidonic acid.

#### Platelet Aggregation

Blood (18 mL) was collected into tubes containing 2 mL of 3.8% sodium citrate and mixed gently. To obtain platelet-rich plasma, the sample was centrifuged at 300*g* at room temperature for 15 minutes. Platelet-rich plasma (2 mL) was further centrifuged at 5500*g* for 6 minutes to obtain platelet-poor plasma. Platelet aggregation was assessed by optical aggregometry (PAP-8E; Bio/Data Corp, Horsham, PA) of the platelet-rich plasma. Before testing, 250 μL of platelet-poor plasma was pipetted into a glass cuvette as a reference standard. Platelet-rich plasma (225 μL) was pipetted into 8 prewarmed (37°C) glass cuvettes with a disposable magnetic stirrer. Agonists (25 μL) were added to platelet-rich plasma and changes in light transmission used to construct aggregation curves.

#### Platelet Activation

Platelet surface P-selectin expression and platelet-monocyte aggregates were measured by flow cytometry. Blood (5 mL) was collected into 50 µL of 1 mmol/L D-phenylalanyl-L-propyl-L-arginine chloromethylketone (Enzo Life Sciences, Exeter, United Kingdom) and immediately aliquoted into Eppendorf tubes prefilled with or without agonist (PAR4-activating peptide, 200 µmol/L; ADP, 10 µmol/L; or arachidonic acid, 5 mmol/L) and the following conjugated monoclonal antibodies: Alexa Fluor647–conjugated CD14, PE (phycoerythrin)-conjugated CD62P, and fluorescein isothiocyanate–conjugated CD42a (Becton-Dickinson). All antibodies were diluted 1:10. Samples were incubated for 20 minutes at room temperature before fixing with 1% paraformaldehyde (P-selectin) or FACS-Lyse (Becton-Dickinson; platelet-monocyte aggregates). All samples were analyzed within 24 hours using an Attune NxT Flow Cytometer (Invitrogen). Data analysis was performed using FlowJo v10 (11 Treestar, OR).

#### Pharmacokinetic Analysis

Samples were collected into 3 mL 1% to 2% ethylenediaminetetraacetic acid for assessment of BMS-986141 and 2.7 mL 3.2% sodium citrate for apixaban. Samples were gently inverted and kept at room temperature for 15 minutes before centrifugation at 1100 to 1500*g* for 10 minutes and then stored at −80°C before analysis.

Plasma BMS-986141 concentrations were determined following treatment using a validated liquid chromatography tandem mass spectrometry method with a lower limit of quantification of 0.050 ng/mL, with an accuracy coefficient of variation of <5% and precision (intra- and inter-assay) coefficients of variation of <10%. Blood samples were collected into 2 mL ethylene diamine tetra-acetic acid tubes (Becton-Dickinson, Cowley, United Kingdom) and placed on wet ice. Within 1 hour of collection, samples were centrifuged at 1200*g* (2–8°C) for 10 minutes. Plasma was decanted and stored at −20°C before analysis.

Plasma apixaban concentrations were determined following treatment as described previously.^22^ Blood samples were collected into 3 mL 3.2% sodium citrate tubes (Becton-Dickinson) and placed on wet ice. Within 1 hour of collection, samples were centrifuged at 1200*g* (2–8°C) for 10 minutes. Plasma was decanted and stored at −20°C before analysis.

#### Histomorphometric Analysis

The porcine aortic strip was removed from each chamber and immediately placed in fixative. Following fixation, the proximal and distal 1 mm of exposed substrate were discarded and the remainder cut into 8 identically sized segments. Individual segments were embedded in paraffin wax from which 4-μm sections were prepared for histomorphometric analysis.

To detect total thrombus area, sections were deparaffinized and rehydrated through a series of xylene and graded alcohols to water. Sections were loaded onto preprogrammed Leica BOND-MAX or BOND RX automated immunostainer. Antigen retrieval was performed by the application of proteinase K (Dako; S3020) for 5 minutes. Endogenous hydrogen peroxide activity was blocked using 3% hydrogen peroxide solution (Leica Microsystems GmbH, Wetzlar, Germany). Sections were then incubated at room temperature for 1 hour with polyclonal rabbit anti-human fibrin(ogen) antibody (1:5000; Dako, Glostrup, Denmark; catalog No. A0080) and monoclonal mouse anti-human CD61 antibody (1:50; Dako; catalog No. M0753). Antigen visualization was performed using a bond polymer refine detection kit (Leica Microsystems GmbH) and treatment with DAB (3,3′-diaminobenzidine) substrate chromagen. Finally, sections were counterstained with a modified direct red protocol.

To examine the effects on fibrin-rich and platelet-rich thrombus formation, endogenous hydrogen peroxide activity was blocked using 3% hydrogen peroxide solution (VWR; Radnor, PA) for 10 minutes and nonspecific binding blocked using 20% normal goat serum (Biosera, Nuaille, France) in tris-buffered saline with 0.01% tween. Sections were then incubated with polyclonal rabbit anti-human fibrin(ogen) antibody (1:50 000) to detect fibrin and CD61 monoclonal mouse anti-human antibody (1:200) to detect platelets. Following washes, and further antigen retrieval with ER1 (citrate with a pH range of 5.9–6.1; Leica Biosystems GmbH, Wetzlar, Germany) goat anti-rabbit peroxidase and goat anti-mouse peroxidase, respectively (1:1000; Abcam, Cambridge, United Kingdom), were applied and the presence of antigen visualized with tyramide Cy3 (1:50; Perkin Elmer, Boston, MA; catalog No. NEL744B001KT) and fluorescein isothiocyanate (1:50; Perkin Elmer, Waltham, MA; catalog No. NEL741B001KT) before nuclear counterstaining with 4′,6-diamidino-2-phenylindole (1:500; Sigma-Aldrich; catalog No. D9542).

Images of stained tissue sections were acquired using a semiautomated slide scanner (Axioscan Z1; Zeiss, Jena, Germany) at ×20 magnification. Separate profiles were created for bright-field (DAB immunohistochemistry with Direct Red counterstain) or 3-channel fluorescence using a halide light source with matched excitation and narrow band pass filters. Images were exported using a 50% resize and cropped to the region of interest, ready for automated analysis. Image analysis was performed by a blinded operator with ImageJ (FIJI, University of Wisconsin),^23^ using a custom script, which was used to batch process images. Briefly, images were processed by detecting the stained regions of porcine aortic strip tissue using thresholding and filtering to measure clot area. Thresholds were determined using positive and negative controls and kept consistent across all images. Representative images of stained porcine aortic strips are shown in Figure II in the Data Supplement.

### Statistical Analysis

Following study completion, the data were cleaned and database locked. Baseline characteristics of volunteers are expressed as percentages for categorical variables and mean±SD for continuous variables. The thrombus area under conditions of high shear stress was taken as the mean of the values from the 2 high-shear chambers. The effect of study drugs was assessed by mean differences for the change from baseline (response to vehicle) and analyzed using 1-way ANOVA followed by Fisher least significant difference test on GraphPad Prism (version 8.0, GraphPad Software; San Diego, CA). Two-sided *P*<0.05 was considered statistically significant.

## Results

Of the 18 recruited volunteers, 15 subjects completed the study in full (Table II in the Data Supplement). Three subjects were withdrawn due to lack of availability or failure to achieve adequate repeated venous cannulation. Pharmacokinetic analysis of the venous effluent confirmed concentrations of BMS-986141 and apixaban in the chamber approximated to the intended target concentrations (Table III in the Data Supplement).

### Platelet Aggregation and Activation

**Figure 1.**
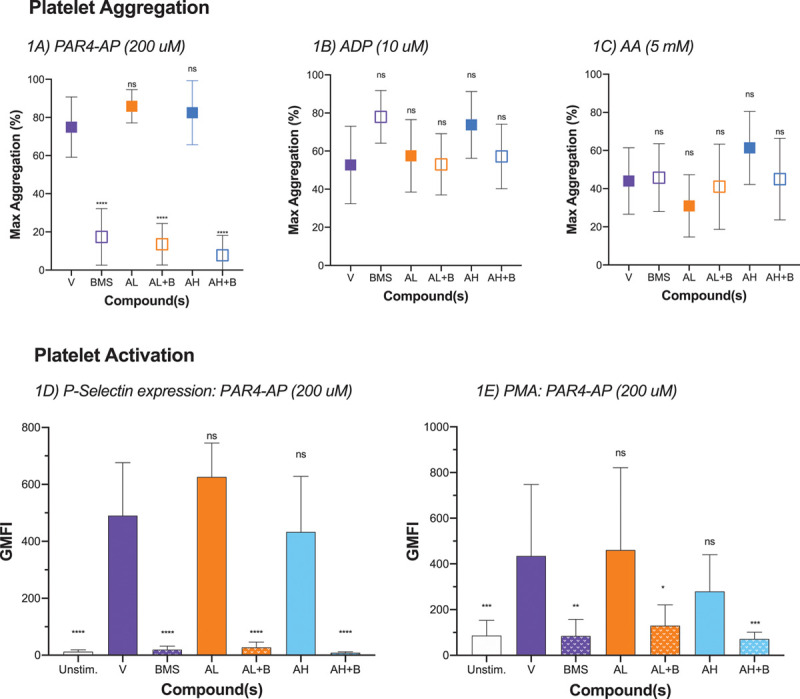
**Effect of compound(s) of platelet aggregation and activation.** n=10 to 15, samples were excluded if samples were hemolyzed or insufficient for further analysis. **A**, Neither vehicle alone (V) nor apixaban alone at high or low dose inhibited platelet aggregation stimulated by PAR4-AP (protease-activated receptor 4 activating peptide). BMS-986141 (BMS/B) alone and in combination with apixaban inhibited platelet aggregation stimulated by PAR4-AP. **B**, Neither BMS nor apixaban inhibited platelet aggregation stimulated by ADP. **C**, Neither BMS nor apixaban inhibited platelet aggregation stimulated by arachidonic acid (AA). **D**, Neither vehicle alone nor apixaban alone at high or low dose inhibited expression of platelet P-selectin stimulated by PAR4-AP. BMS alone and in combination with apixaban inhibited expression of platelet P-selectin stimulated by PAR4-AP. **E**, Neither vehicle alone nor apixaban alone at high or low dose inhibited platelet-monocyte aggregate (PMA) formation by PAR4-AP. BMS alone and in combination with apixaban inhibited PMA formation stimulated by PAR4-AP. BMS, 400 ng/mL; AL, 20 ng/mL; AH, 80 ng/mL; PAR4-AP, 200 µmol/L; ADP, 10 µmol/L; AA, 5 µmol/L. Arbitrary units ×10^3^ for P-selectin expression and ×10^2^ for platelet-monocyte aggregates. Comparisons vs vehicle: ns, *P*>0.05. AH indicates high-dose apixaban; AL, low-dose apixaban; GMFI, geometric mean fluorescent intensity; Max, maximum; and ns, nonsignificant. *P<0.03, **P<0.02, ***P<0.01, ****P<0.0001.

As anticipated, ADP, arachidonic acid, and PAR4-activating peptide stimulation caused platelet aggregation. BMS-986141 had no effect on platelet aggregation stimulated by ADP (*P*>0.08) or arachidonic acid (*P*>0.7) but inhibited PAR4-activating peptide–stimulated platelet aggregation (*P*<0.0001). In contrast, apixaban alone had no effect on ADP (*P*>0.05 for both doses), arachidonic acid (*P*>0.05 for both doses), or PAR4-activating peptide–stimulated platelet aggregation (*P*>0.05 for both doses; Figure 1; Table IV in the Data Supplement). Apixaban did not interfere with inhibition of PAR4-activating peptide–stimulated platelet aggregation by BMS-986141 (*P*<0.0001).

PAR4-activating peptide stimulation increased platelet P-selectin expression and platelet-monocyte aggregation (Figure 1; Table IV in the Data Supplement). BMS-986141 reduced both PAR4-activating peptide–stimulated P-selectin expression (*P*<0.0001) and platelet-monocyte aggregation (*P*<0.012). Apixaban had no effect in isolation and did not interfere with PAR4 inhibition by BMS-986141.

### Ex Vivo Thrombus Formation

#### High-Shear Chamber

**Figure 2.**
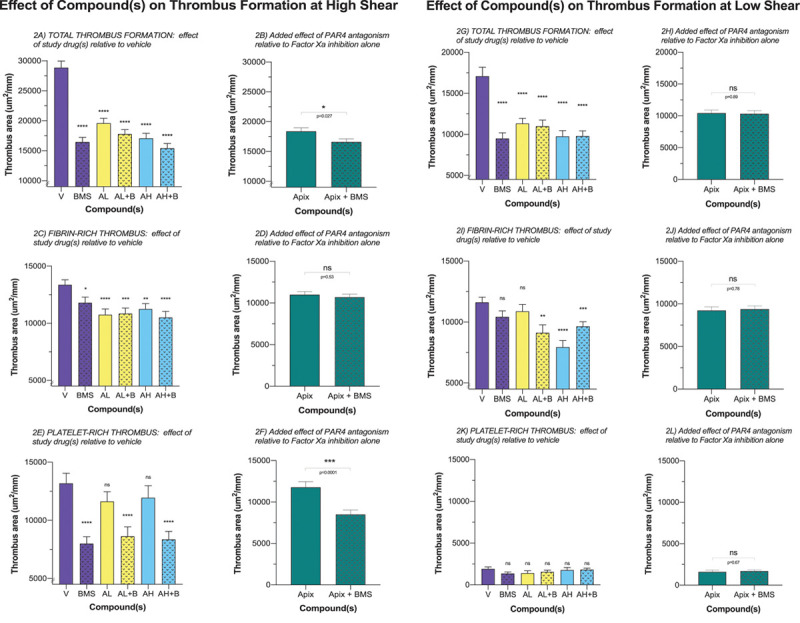
**Effect of compound(s) on thrombus formation in the high shear and low shear stress chamber.** n=15. **A**, All drugs reduced total thrombus formation at high shear. **B**, Overall, the addition of BMS-986141 (BMS/B) to apixaban (Apix) caused a further reduction of total thrombus formation. **C**, Apix reduced fibrin-rich thrombus formation; BMS had a smaller effect on fibrin-rich thrombus formation. **D**, The addition of BMS to Apix did not affect the overall reduction in fibrin-rich thrombus formation caused by Apix at high shear. **E**, Apix at low and high doses had no effect on platelet-rich thrombus formation, whereas BMS markedly reduced platelet-rich thrombus formation. **F**, The addition of BMS to Apix caused a further reduction of platelet-rich thrombus formation. **G**, All drugs reduced total thrombus formation at low shear. **H**, Overall, the addition of BMS to Apix had no effect on total thrombus formation. **I**, BMS and low-dose Apix had no effect on fibrin-rich thrombus formation, whereas high-dose Apix and the combination of BMS to low- or high-dose Apix led to a reduction in fibrin-rich thrombus area. **J**, The addition of BMS to Apix did not lead to further reduction in fibrin-rich thrombus area. **K**, None of the drugs had any effect on platelet-rich thrombus formation. **L**, The addition of BMS to Apix did have any additional effect on platelet-rich thrombus formation. BMS, 400 ng/mL; AL, 20 ng/mL; AH, 80 ng/mL. Comparisons vs vehicle: ns, *P*>0.05. AH indicates high-dose apixaban; AL, low-dose apixaban; Apix, low-dose Apix and high-dose Apix; Apix+BMS, low-dose Apix+BMS and high-dose Apix+BMS; ns, nonsignificant; PAR4, protease-activated receptor; and V, vehicle alone. *P<0.05, **P<0.004, ***P<0.0002, ****P<0.0001.

Under conditions of high shear stress, BMS-986141 reduced total thrombus area by 43.4% and apixaban by up to 40.9% in a dose-dependent manner (Figure 2A). Overall, the addition of BMS-986141 with apixaban caused a further reduction in total thrombus formation by 9.65% (*P*=0.027; Figure 2B; Table V in the Data Supplement).

For fibrin-rich thrombus, there was a modest reduction in thrombus area by BMS-986141 (11.7%; *P*=0.018) but a more prominent reduction with apixaban (19.6% and 15.8%; *P*<0.0001; at 20 and 80 ng/Ml, respectively; Figure 2C; Table VI in the Data Supplement). There was no discernible additive effect of combining apixaban with BMS-986141 on reductions in fibrin-rich thrombus area (Figure 2D; Table VI in the Data Supplement).

For platelet-rich thrombus, there was a marked reduction in thrombus area with BMS-986141 (39.3%; *P*<0.0001) but no effect with apixiban (11.8% and 9.35%; *P*=0.16 and 0.28; at 20 and 80 ng/mL, respectively; Figure 2E; Table VI in the Data Supplement). The addition of BMS-986141 to apixaban caused a further substantial reduction in thrombus area (27.8%; *P*=0.0001; Figure 2F; Table VI in the Data Supplement).

#### Low-Shear Chamber

Under conditions of low shear, BMS-986141 reduced total thrombus area by 44.4% and apixaban by up to 42.9% in a dose-dependent manner (Figure 2G). No additive effect was detected by combining BMS-986141 with apixaban (0.9%; *P*=0.89; Figure 2H). Apixaban reduced fibrin-rich thrombus area in a dose-dependent manner (6.4% and 31.6%; *P*=0.32 and <0.0001; at low and high doses, respectively). Conversely BMS-986141 alone did not reduce fibrin-rich thrombus formation (*P*>0.05; Figure 2I). Furthermore, no additive effect was detected on combining BMS-986141 with apixaban (Figure 2J; Table VI in the Data Supplement). There was little effect on platelet-rich thrombus area under conditions of low shear stress regardless of drug or drug combination (Figure 2K). Moreover, the addition of BMS-986141 to apixaban had no effect on the reduction of platelet-rich thrombus area (Figure 2L; Table VI in the Data Supplement).

## Discussion

In a double-blind randomized controlled study, we assessed the effects of PAR4 antagonism with BMS-986141 and factor Xa inhibition with apixaban, alone and in combination, on human platelet aggregation, activation, and ex vivo thrombus formation. Consistent with its known pharmacological actions, BMS-986141 markedly inhibited PAR4-mediated platelet aggregation and activation, and this was unaffected by concomitant factor Xa inhibition with apixaban. Importantly, when used in combination with apixaban, BMS-986141 produced an additional modest reduction in total thrombus area, which was attributable to inhibition of platelet-rich thrombus, especially under conditions of high shear stress. This suggests a potential additive benefit of combined factor Xa and PAR4 antagonism, which could provide a novel therapeutic antithrombotic strategy for the prevention of cardiovascular events that requires further exploration.

Using the venous effluent from the perfusion chamber, we confirmed that we had achieved high concentrations of BMS-986141 in the perfusion chamber, and this was predicted to have a near maximal inhibitory effect on platelet PAR4 receptors. Indeed, at this concentration, we confirmed that BMS-986141 selectively inhibited PAR4-mediated platelet aggregation and activation, whereas apixaban had no discernible effect. Moreover, BMS-986141, but not apixaban, caused a clear and marked reduction in platelet-rich thrombus formation, especially under conditions of high shear stress. This confirms our previous findings^19^ that PAR4 antagonism is an effective antiplatelet agent that would be anticipated to inhibit and to reduce arterial thrombosis.

We have previously demonstrated that bivalirudin—a direct thrombin inhibitor—and JNJ-64179375—an exosite 1 thrombin inhibitor—inhibit fibrin-rich thrombus formation in this ex vivo model of acute arterial injury.^19^ Consistent with their anticoagulant action, they inhibited fibrin-rich thrombus formation in a dose-dependent manner. Apixaban (10–160 ng/mL) has also been studied in perfusion chamber models and has similarly demonstrated dose-dependent reductions in thrombus area.^24^ We have here confirmed these findings and demonstrated a dose-dependent reduction in thrombus formation and specifically fibrin-rich thrombus as would be anticipated by its mode of action.

At very high doses, apixaban can reduce platelet-rich thrombus formation,^24^ and this may be through the inhibition of thrombin-mediated platelet activation. We, therefore, wanted to establish whether PAR4 antagonism would be efficacious at clinically relevant plasma apixaban concentrations, due to concerns that factor Xa inhibition would completely inhibit thrombin-mediated platelet activation. If this were to occur, it would be challenging for PAR4 antagonism with BMS-986141 to produce any additional antithrombotic effect. To address this concern, we selected plasma apixaban concentrations of 20 and 80 ng/mL to reflect median average exposures expected with dosing of 2.5 mg QD or 5 mg BID. At these concentrations, we observed the anticipated dose-dependent reduction in total and fibrin-rich thrombus area in our perfusion chamber model, confirming its anticipated efficacy. However, we were also able to observe that the addition of PAR4 antagonism with BMS-986141 caused a further reduction in thrombus area, which was particularly marked for platelet-rich thrombus area. This would, therefore, suggest that at therapeutic doses, apixaban does not inhibit thrombin generation enough to prevent activation of PAR4 receptors so that thrombin-mediated platelet activation and aggregation continues to occur. Combined PAR4 antagonism and factor Xa inhibition, therefore, has potential additive therapeutic efficacy that merits further clinical investigation to reduce arterial thrombosis and atherothrombotic events.

There have been several previous studies examining the effects of combined antiplatelet and anticoagulation therapies in patients at risk of recurrent cardiovascular events. In the COMPASS trial (Cardiovascular Outcomes for People Using Anticoagulation Strategies), the combination of a factor Xa inhibitor rivaroxaban with aspirin was more efficacious than either drug alone in reducing recurrent cardiovascular events.^11^ However, in the COMPASS, APPRAISE-2 (Apixaban With Antiplatelet Therapy After Acute Coronary Syndrome) and AUGUSTUS (Antithrombotic Therapy After Acute Coronary Syndrome or PCI in Atrial Fibrillation) trials, the combination of antiplatelet and anticoagulant therapies had an important bleeding liability.^12,25^ The challenge is, therefore, to develop efficacious combination therapies without substantial bleeding hazard. To date, the commonest antiplatelet therapy tested has been aspirin although P2Y12 receptor antagonists and triple therapies have also been assessed. The gastrointestinal and broad spectrum of action of aspirin and P2Y12 receptor antagonists can increase bleeding risk when used in combination with an anticoagulant treatment. More selective pathway inhibition with PAR4 antagonism may present an opportunity to reduce atherothrombotic events while limiting bleeding risk. Indeed, prior work in cynomolgus monkeys suggests that PAR4 antagonism has a lower bleeding risk than P2Y12 antagonism with clopidogrel.^7^ The present model does not allow for the assessment of bleeding risk, and, while we have demonstrated the potential for improved efficacy, both treatment efficacy and potential bleeding liabilities will need to be established in subsequent clinical trials. However, our previous work with a closely related analogue of the PAR4 antagonist was well tolerated with no major side effects or serious adverse events.^5^

In this present study, BMS-986141 also reduced total thrombus formation in conditions of low shear stress. This was an unexpected finding since in our previous studies of a similar but orally ingested PAR4 antagonist,^5^ PAR4 antagonism did not have a discernible effect on low shear total thrombus formation. This may be due to differences between compounds or experimental setup (extracorporeal addition of PAR4 antagonist verses oral ingestion). It also likely reflects the greater variability of the low shear stress thrombus findings as these are derived from a single chamber compared with the mean of the 2 high-shear chambers. Moreover, BMS-986141 had only modest effects on the components of platelet-rich and fibrin-rich thrombus areas that seemed out of keeping with the findings from the total thrombus area. Given this lack of consistency, we feel this likely represents a chance finding of uncertain significance.

Although this was a double-blind randomized controlled study and the primary end point was measured by blinded assessors, we acknowledge several limitations. First, we explored the effect of BMS-986141 and apixaban through extracorporeal administration of these drugs. This may have caused some alterations in the behavior of these compounds although our pharmacokinetic data confirm we achieved the approximate target plasma concentrations for each compound. Second, we required a vehicle to ensure solubility of BMS-986141. We maintained the same concentration of vehicle in all 6 infusions to minimize any potential bias, although we cannot exclude the possibility that the vehicle may have influenced the pharmacodynamics of both apixaban and BMS-986141 in our model. However, we confirmed the anticipated selective antiplatelet actions of BMS-986141 on platelet activation and aggregation in the effluent of the chamber, as well as the anticipated effects of BMS-986141 and apixaban on platelet-rich high-shear and fibrin-rich low shear stress thrombus formation. Third, we did not compare PAR4 inhibition in combination with apixaban to contemporary antiplatelet agents with apixaban. While this certainly would be of interest, our aim was to establish whether the effects of apixaban would limit the ability of PAR4 antagonism to reduce thrombus formation. This narrow focus, therefore, allows us to answer this specific question with confidence. Finally, we did not explore the dose-response relationship for BMS-986141 in this study, but our primary focus was to demonstrate whether there was added efficacy of PAR4 antagonism when added to clinically relevant doses of the factor Xa inhibitor apixaban.

In conclusion, we have demonstrated that PAR4 antagonism when used in combination with factor Xa inhibition causes an additive reduction in human thrombus formation, especially for platelet-rich thrombus under conditions of high shear stress. This suggests the potential for additive benefit of combination therapy in reducing arterial thrombosis and atherothrombotic clinical events.

## Acknowledgments

We are grateful to the Queen’s Medical Research Institute (Edinburgh, United Kingdom) for their support and expertise in conducting this study. Edinburgh Clinical Research Facility is supported by the National Health Service Research Scotland through the National Health Service Lothian Health Board.

## Sources of Funding

This study was funded by Bristol-Myers Squibb.

## Disclosures

D.E. Newby holds educational grants and has received consultancy from Bristol-Myers Squibb and Pfizer. V. Perera, S.M. Garonzik, B. Murthy, J.G. Everlof, R. Aronson, and J. Luettgen are employed by Bristol-Myers Squibb. The other authors report no conflicts.

## Supplementary Material


