# Supplementary material for: Antithrombotic Effects of Combined PAR (Protease-Activated Receptor)-4 Antagonism and Factor Xa Inhibition
Source: Arterioscler Thromb Vasc Biol. 2020 Sep 10;40(11):2678–85. doi: 10.1161/ATVBAHA.120.314960 (PMC7571844; doi:10.1161/ATVBAHA.120.314960)
Supplement: Supplementary file 1 [file atv-40-2678-s001.pdf]

SUPPLEMENTAL MATERIALS

Antithrombotic effects of combined protease-activated receptor-4 antagonism and factor Xa inhibition

Authors:

Mohammed N Meah (MB ChB)[a], Jennifer Raftis (PhD) [a], Simon J. Wilson (PhD) [a], Vidya Perera (PhD)[b], Samira M Garonzik (MS; PharmD)[b], Bindu Murthy (PharmD)[b], J Gerry Everlof (MS)[c], Ronald Aronson (MD)[b], Joseph Luettgen (BS)[c], David E Newby (DSc)[a]

[a]British Heart Foundation Centre for Cardiovascular Science, University of Edinburgh, Edinburgh, UK

[b]Bristol-Myers Squibb, Princeton Pike Facility, Princeton, New Jersey, USA

[c]Brisol-Myers Squibb, Lawrenceville Facility, Princeton, New Jersey, USA

Supplemental Figures

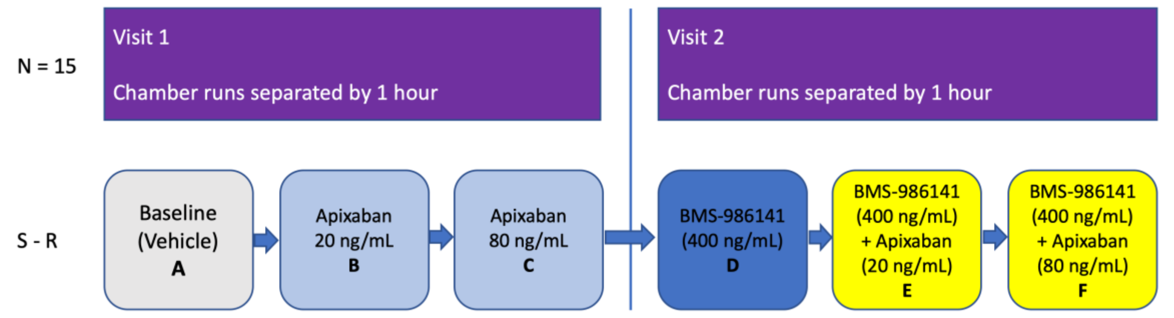

Figure I: Summary of study design
Total of 15 participants completed 6 perfusion chamber studies over two days in a randomised sequence.
S – Screening, R – Randomisation.

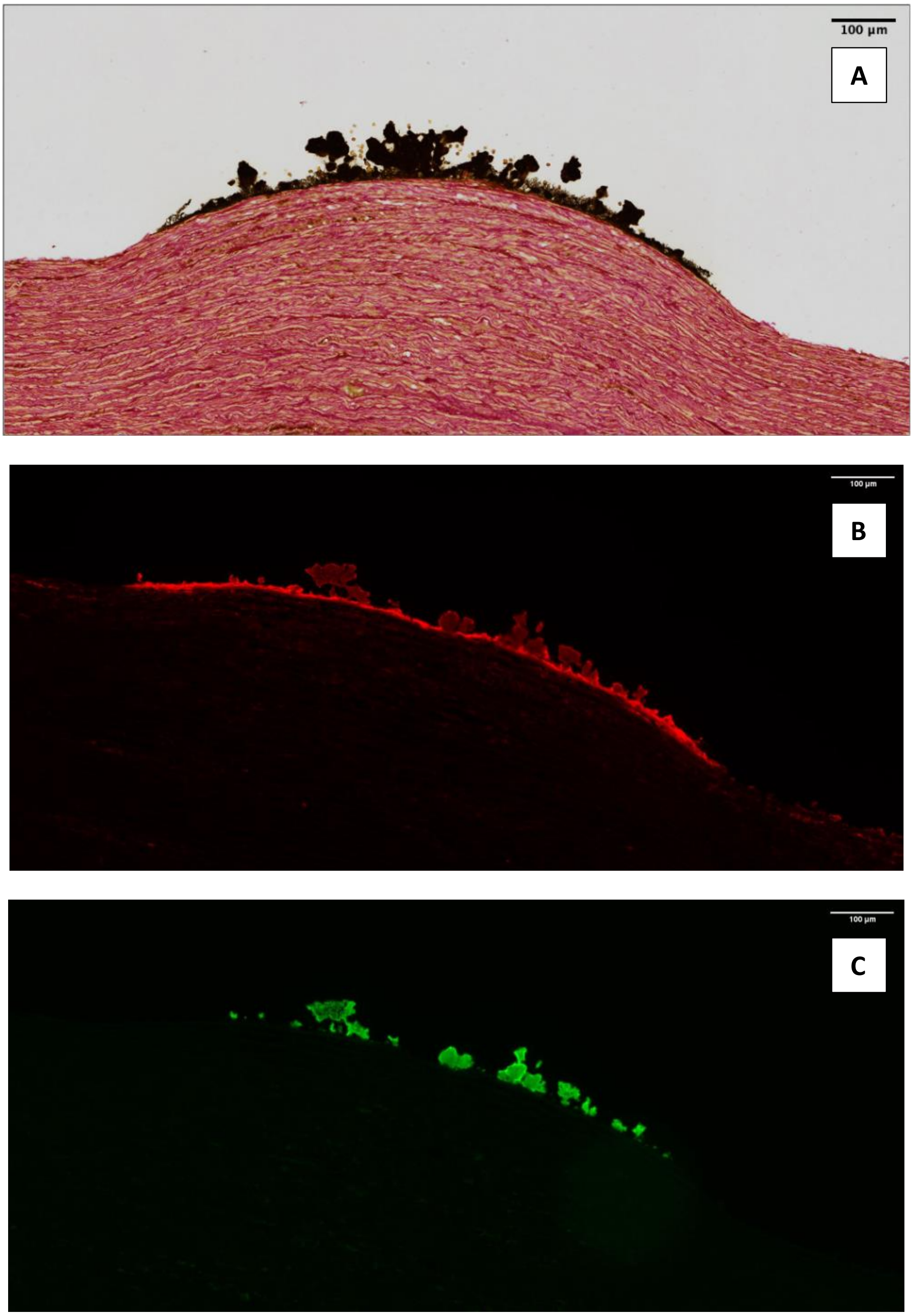

Figure II: Representative images of porcine aorta.
[A] Representative image of porcine aorta exposed to human blood with polyclonal goat antihuman fibrin antibody and CD61 monoclonal mouse antihuman antibody to allow quantification of total thrombus area.
[B] Representative image of porcine aorta exposed to human blood with polyclonal goat antihuman fibrin antibody and CD61 monoclonal mouse antihuman antibody to allow quantification of fibrin-rich thrombus area (counterstained with cyanine 3).
[C] Representative image of porcine aorta exposed to human blood with polyclonal goat antihuman fibrin antibody and CD61 monoclonal mouse antihuman antibody to allow quantification of platelet-rich thrombus area (counterstained with fluorescein isothiocyanate).

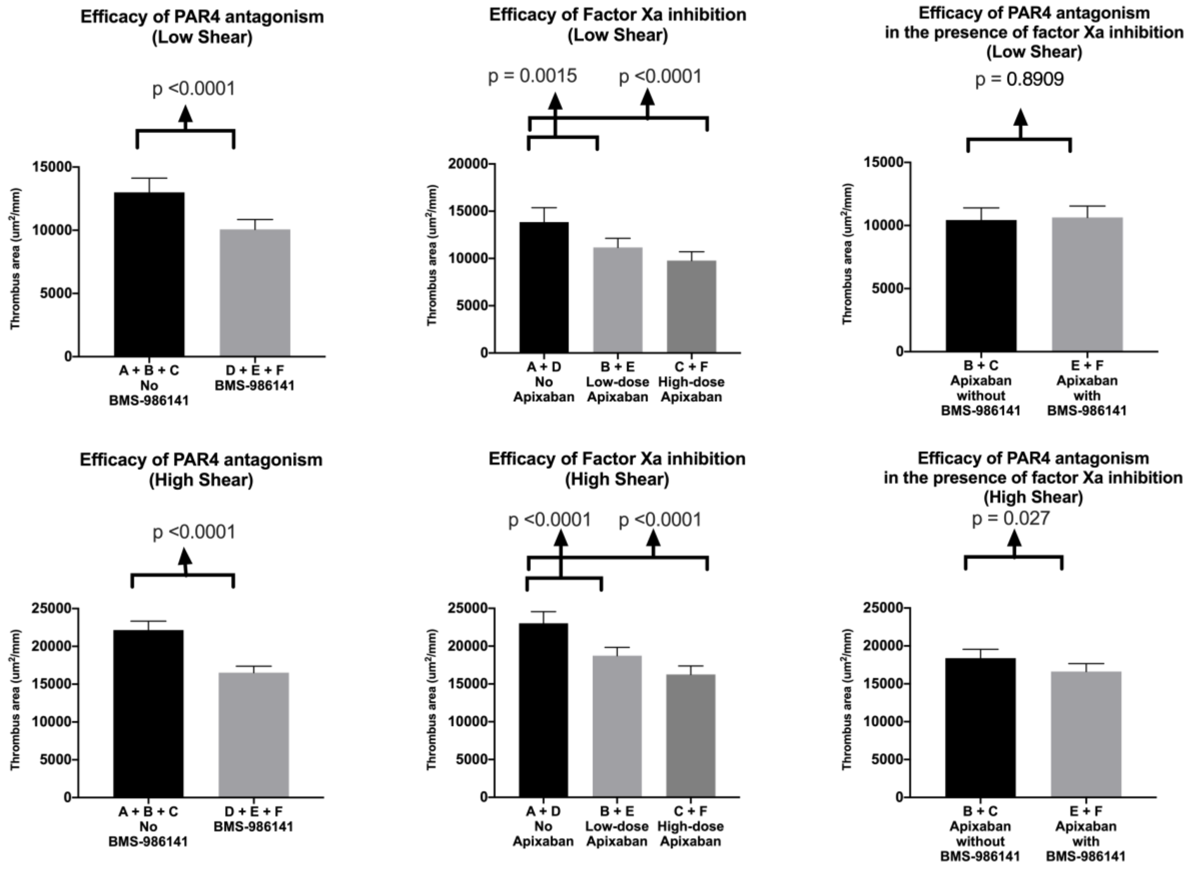

Figure III: Total Thrombus Area in the *Ex-Vivo* Perfusion Chamber
Efficacy of compounds at reducing total thrombus area in conditions of low-shear and high-shear stress. All compound(s) reduced total thrombus area at both high and low-shear stress conditions. The addition of BMS-986141 to apixaban led to further reduction in total thrombus area in conditions of high-shear stress.

(A)- Vehicle, (B)- low dose apixaban, (C)- high dose apixaban, (D)- BMS-986141, (E)- low-dose apixaban and BMS-986141, (F)- high-dose apixaban and BMS-986141.

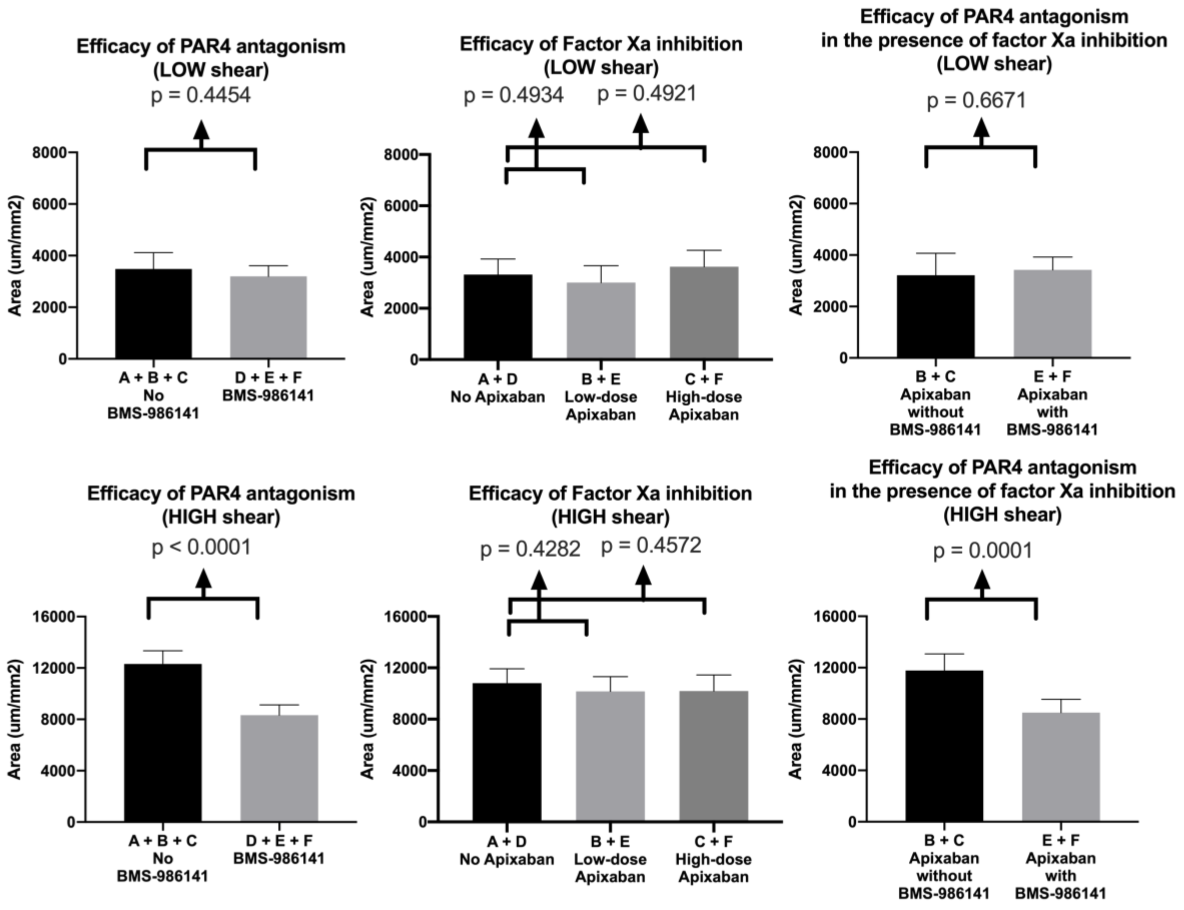

Figure IV: Platelet-rich Thrombus Area in the *Ex-Vivo* Perfusion Chamber
Efficacy of compounds at reducing platelet-rich thrombus area in conditions of low-shear and high-shear stress. Compound(s) had no discernible effect in conditions of low shear. At high shear BMS-986141 but NOT apixaban, caused a reduction in platelet-rich thrombus area. The addition of BMS to apixaban caused further reductions in platelet-rich thrombus area.

(A)- Vehicle, (B)- low dose apixaban, (C)- high dose apixaban, (D)- BMS-986141, (E)- low dose apixaban and BMS-986141, (F)- high dose apixaban and BMS-986141.

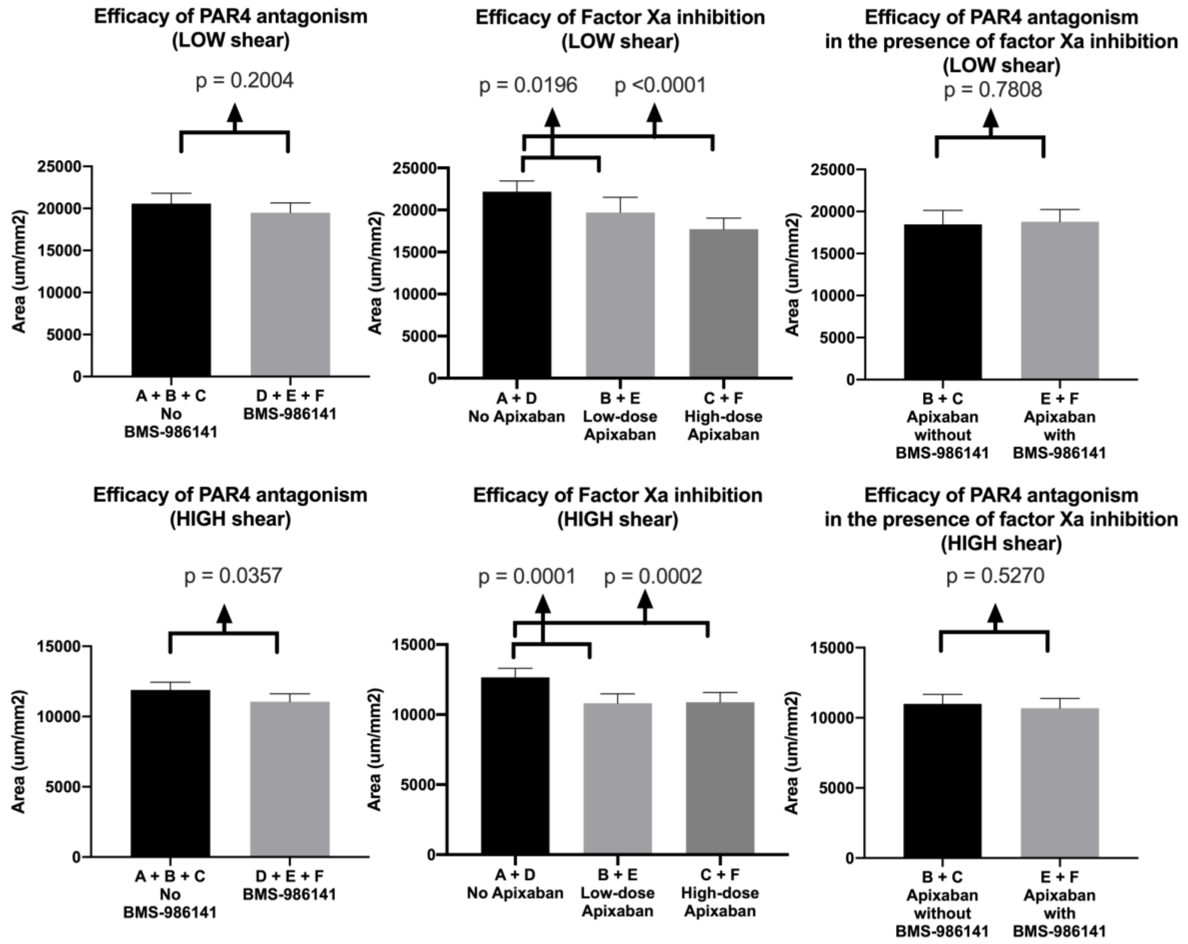

Figure V: Fibrin-rich Thrombus Area in the *Ex-Vivo* Perfusion Chamber
Efficacy of compounds at reducing fibrin-rich thrombus area in conditions of low-shear and high-shear stress. BMS-986141 had no effect on fibrin-rich thrombus area in conditions of low shear but caused a modest reduction in conditions of high-shear. Apixaban caused reductions in fibrin-rich thrombus area in both low and high-shear conditions in a dose-dependent manner. The addition of BMS-986141 to apixaban had no discernible additive effect.

(A)- Vehicle, (B)- low dose apixaban, (C)- high dose apixaban, (D)- BMS-986141, (E)- low dose apixaban and BMS-986141, (F)- high dose apixaban and BMS-986141.

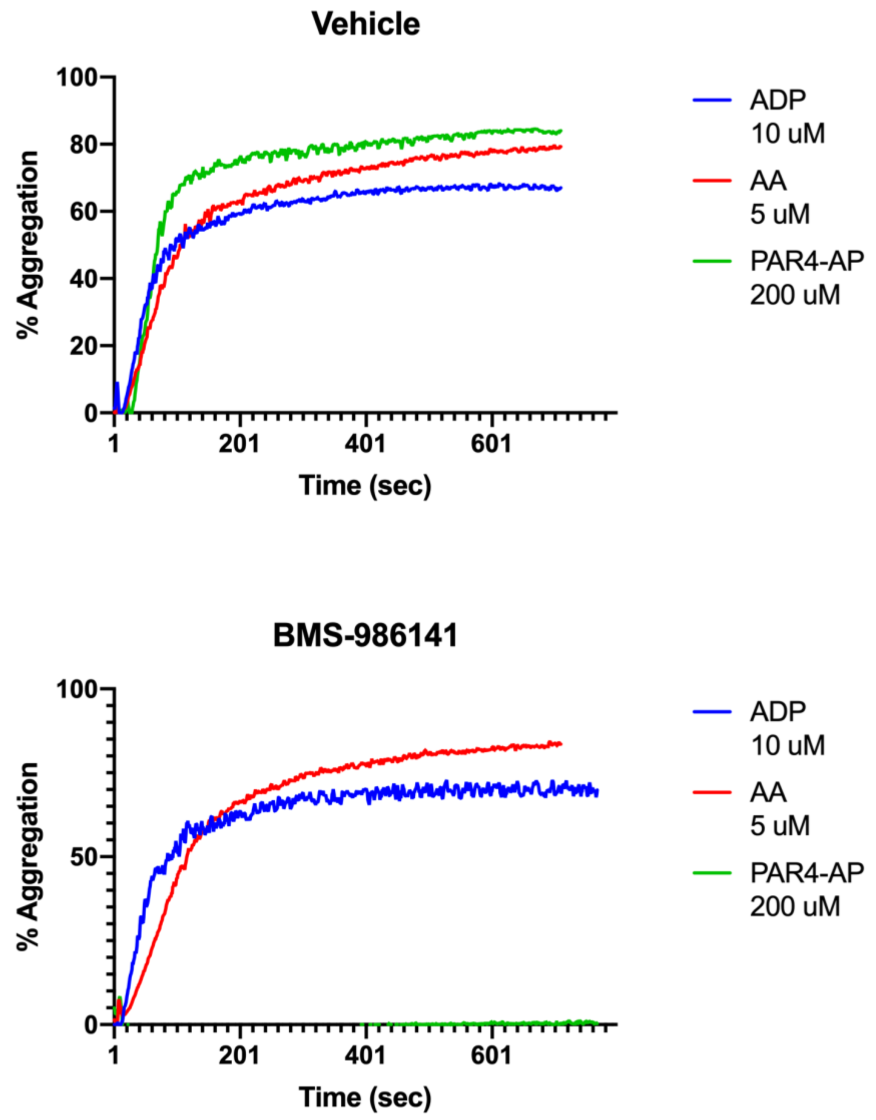

Figure VI: Representative aggregometry curves for Vehicle and BMS-986141

Supplemental Tables

Table I: Planned Perfusion Sequences

No of participants	Perfusion Sequence 1	Perfusion Sequence 2	Perfusion Sequence 3	Perfusion Sequence 4	Perfusion Sequence 5	Perfusion Sequence 6
**N= 3**	A	B	C	D	E	F
**N= 2**	B	C	D	E	F	A
**N= 2**	C	D	E	F	A	B
**N= 3**	D	E	F	A	B	C
**N= 2**	E	F	A	B	C	D
**N= 3**	F	A	B	C	D	E

(A)- Placebo/vehicle, (B)- low-dose apixaban, (C)- high-dose apixaban, (D)- BMS-986141, (E)- BMS-986141 and low-dose apixaban, (F)- BMS-986141 and high-dose apixaban.

Table II: Baseline characteristics of study participants

Men	8 (53)
Age (years)	29.5±6.1
Body-mass index (kg/m^2)	24.4±3.5
Haemoglobin (g/dL)	145±12
Platelet count (x10^9 /L)	260±41
Activated partial thromboplastin time (s)	29.8±4.1
Prothrombin time (s)	11.9±0.74

n (%); mean±standard deviation.

Table III: Effluent Plasma Concentrations of Apixiban and BMS-986141

Study Code	Study Drug and Target Concentration	Plasma Concentration (ng/mL)
**A**	Vehicle	-
**B**	Apixaban, 20 ng/mL	15.8±2.4
**C**	Apixaban, 80 ng/mL	72.3±18.7
**D**	BMS-986141, 400 ng/mL	285.0±75.1
**E**	Apixaban, 20 ng/mL	16.8±4.8
	BMS-986141, 400 ng/mL	289.9±68.2
**F**	Apixaban, 80 ng/mL	60.8±25.1
	BMS-986141, 400 ng/mL	293.3±76.6

mean±standard deviation

Table IV: Platelet studies			
**Comparison of max platelet aggregation in presence of ADP**			
**Comparators**	**Mean difference**	**95% Confidence Interval**	**Adjusted *P*-value**
**A vs B**	-4.765	-26.32 to 16.79	0.6635
**A vs C**	-21.02	-42.57 to 0.5361	0.0559
**A vs D**	-19.97	-42.40 to 2.461	0.0807
**A vs E**	-0.3042	-21.49 to 20.89	0.9775
**A vs F**	-4.465	-25.65 to 16.72	0.6784
**Comparison of max platelet aggregation in presence of AA**			
**Comparators**	**Mean difference**	**95% Confidence Interval**	**Adjusted *P*-value**
**A vs B**	13.08	-8.474 to 34.63	0.2331
**A vs C**	-17.36	-38.91 to 4.194	0.1139
**A vs D**	4.261	-18.17 to 26.69	0.7085
**A vs E**	2.981	-18.21 to 24.17	0.7819
**A vs F**	-0.9936	-22.18 to 20.20	0.9265
**Comparison of max platelet aggregation in presence of PAR4-AP**			
**Comparators**	**Mean difference**	**95% Confidence Interval**	**Adjusted *P*-value**
**A vs B**	-10.93	-32.48 to 10.63	0.3189
**A vs C**	-7.576	-29.13 to 13.98	0.4893
**A vs D**	64.62	42.19 to 87.05	<0.0001
**A vs E**	61.43	40.24 to 82.62	<0.0001
**A vs F**	67.20	46.01 to 88.39	<0.0001
**Comparison of p-selectin expression in presence of PAR4-AP**			
**A vs D**	470648	319694 to 621602	<0.0001
**A vs E**	462512	311558 to 613466	<0.0001
**A vs F**	481773	330819 to 632726	<0.0001
**C vs E**	-8136	-159089 to 142818	0.9144
**C vs F**	11125	-139829 to 162079	0.8831
**Comparison of platelet-monocyte aggregates in presence of PAR4-AP**			
**A vs D**	35036	7706 to 62366	0.0127
**A vs E**	30586	3256 to 57916	0.0288
**A vs F**	36398	9068 to 63728	0.0098
**C vs E**	-4450	-31780 to 22880	0.7464
**C vs F**	1362	-25968 to 28692	0.9211
(A)- Placebo/vehicle, (B)- low-dose apixaban, (C)- high-dose apixaban, (D)- BMS-986141, (E)- BMS-986141 and low-dose apixaban, (F)- BMS-986141 and high-dose apixaban. ADP – Adenosine diphosphate, AA – arachidonic acid, PAR4-AP – Protease-Activated Receptor 4 agonist peptide			

Table V: Total Thrombus Area				
**Comparison of Total Thrombus Area (LOW-SHEAR STRESS)**				
**Comparators**	**Percentage difference (%)**	**Mean difference (μm2/mm)**	**95% Confidence Interval**	**Adjusted *P*-value**
A vs B	-33.71	5765	3521 to 8008	<0.0001
A vs C	-42.92	7340	5267 to 9414	<0.0001
A vs D	-44.44	7600	5411 to 9789	<0.0001
A vs E	-35.68	6101	3858 to 8344	<0.0001
A vs F	-42.67	7297	5175 to 9418	<0.0001
**Efficacy of PAR4 antagonism ***	-22.59	2937	1616 to 4259	<0.0001
**Efficacy of low-dose apixaban †**	-19.38	2685	1026 to 4344	0.0015
**Efficacy of high-dose apixaban ‡**	-29.39	4072	2515 to 5628	<0.0001
**Efficacy of combination §**	-0.9	94.52	-1553 to 1742	0.8909
**Comparison of Total Thrombus Area (HIGH-SHEAR STRESS)**				
A vs B	-32.13	9278	6924 to 11632	<0.0001
A vs C	-40.92	11816	9403 to 14228	<0.0001
A vs D	-43.44	12544	10152 to 14936	<0.0001
A vs E	-38.38	11081	8663 to 13499	<0.0001
A vs F	-46.52	13432	11019 to 15845	<0.0001
**Efficacy of PAR4 antagonism ***	-25.47	5644	4228 to 7059	<0.0001
**Efficacy of low-dose apixaban †**	-18.58	4277	2565 to 5989	<0.0001
**Efficacy of high-dose apixaban ‡**	-29.40	6767	5035 to 8499	<0.0001
**Efficacy of combination §**	-9.65	1774	18.02 to 3529	0.027

***** [Vehicle + apixaban low-dose + apixaban high-dose] **VS** [BMS + BMS & low-dose apixaban + BMS & high-dose apixaban]

† [Vehicle + BMS] **VS** [low-dose apixaban + BMS & low-dose apixaban]

‡ [Vehicle + BMS] **VS** [high-dose apixaban + BMS & high-dose apixaban]

§ [Apixaban low-dose + Apixaban high-dose] **VS** [BMS & low-dose apixaban + BMS & high-dose apixaban].

(A)- Placebo/vehicle, (B)- low-dose apixaban, (C)- high-dose apixaban, (D)- BMS-986141, (E)- BMS-986141 and low-dose apixaban, (F)- BMS-986141 and high-dose apixaban.

Table VI: Platelet-rich and Fibrin-rich Thrombus Area								
	**Comparison of Platelet-rich Thrombus Area (LOW-SHEAR STRESS)**				**Comparison of Fibrin-rich Thrombus Area (LOW-SHEAR STRESS)**			
**Comparators**	**Percentage difference (%)**	**Mean difference (μm2/mm)**	**95% Confidence Interval**	**Adjusted *P*-value**	**Percentage difference (%)**	**Mean difference (μm2/mm)**	**95% Confidence Interval**	**Adjusted *P*-value**
A vs B	-27.01	1038	-302.2 to 2378	0.1286	-6.42	745.9	-721.4 to 2213	0.3181
A vs C	-7.44	285.4	-975.4 to 1546	0.6565	-31.62	3673	2307 to 5039	<0.0001
A vs D	-28.83	1108	-90.44 to 2306	0.0699	-10.23	1188	-124.6 to 2501	0.0759
A vs E	-18.21	699.5	-503.7 to 1903	0.2537	-21.52	2500	1169 to 3831	0.0003
A vs F	-4.37	167.7	-1004 to 1339	0.7784	-16.96	1970	663.2 to 3277	0.0032
**Efficacy of PAR4 antagonism ***	-8.15	284.1	-445.4 to 1014	0.4454	-5.32	546.9	-290.7 to 1385	0.2004
**Efficacy of low-dose apixaban †**	-9.41	311.8	-588.1 to 1212	0.4934	-11.10	1231	197.4 to 2264	0.0196
**Efficacy of high-dose apixaban ‡**	-9.26	-307.1	-1172 to 557.9	0.4921	-20.05	2222	1229 to 3216	<0.0001
**Efficacy of combination §**	-6.34	-203.4	-1131 to 724.6	0.6671	-1.65	-152.8	-1230 to 924.3	0.7808
	**Comparison of Platelet-rich Thrombus Area (HIGH-SHEAR STRESS)**				**Comparison of Fibrin-rich Thrombus Area (HIGH-SHEAR STRESS)**			
A vs B	-11.82	1558	-627.3 to 3743	0.1621	-19.57	2616	1321 to 3911	<0.0001
A vs C	-9.35	1233	-989.6 to 3456	0.2765	-15.82	2114	808.2 to 3420	0.0015
A vs D	-39.32	5185	2991 to 7379	<0.0001	-11.71	1565	264.8 to 2865	0.0184
A vs E	-34.49	4547	2335 to 6760	<0.0001	-18.82	2516	1212 to 3819	0.0002
A vs F	-36.54	4819	2581 to 7056	<0.0001	-21.32	2850	1544 to 4156	<0.0001
**Efficacy of PAR4 antagonism ***	-32.33	3979	2653 to 5306	<0.0001	-7.01	834.1	55.91 to 1612	0.0357
**Efficacy of low-dose apixaban †**	-5.96	643.3	-955.8 to 2242	0.4282	-14.70	1860	917.8 to 2803	0.0001
**Efficacy of high-dose apixaban ‡**	-5.83	629.2	-991.8 to 2250	0.4572	-14.04	1776	828.6 to 2724	0.0002
**Efficacy of combination §**	-27.83	3280	1622 to 4938	0.0001	-2.85	313.1	-657.3 to 1284	0.5270

***** [Vehicle + apixaban low-dose + apixaban high-dose] **VS** [BMS + BMS & low-dose apixaban + BMS & high-dose apixaban], † [Vehicle + BMS] **VS** [low-dose apixaban + BMS & low-dose apixaban], ‡ [Vehicle + BMS] **VS** [high-dose apixaban + BMS & high-dose apixaban], § [Apixaban low-dose + Apixaban high-dose] **VS** [BMS & low-dose apixaban + BMS & high-dose apixaban]. (A)- Placebo/vehicle, (B)- low-dose apixaban, (C)- high-dose apixaban, (D)- BMS-986141, (E)- BMS-986141 and low-dose apixaban, (F)- BMS-986141 and high-dose apixaban.

Major Resources Table:

Antibodies

Target antigen	Vendor or Source	Catalog #	Working concentration
Fibrinogen (Rabbit)	DAKO	A0080	1.2 μg/mL (1:5000)
CD61 (Mouse)	Dako	M0753	1.28 μg/mL 1:50
Normal goat serum	Bio sera	GO605/500	1:5
Goat anti Rabbit Peroxidase	Abcam	Ab7171	1:1000
Goat anti-Mouse Peroxidase	Abcam	Ab6823	1:1000
DAPI	Sigma-Aldrich	D9542	5 μg/mL 1:500
Tyrimide Cy3	Perkin Elmer	NEL 744B001KT	1:50
Tyrimide FITC	Perkin Elmer	NEL 741B00KT	1:50

Other

Description	Source / Repository
Proteinase k	DAKO: S3020
Refine kit	Leica biosystems: DS9800

DOI [to be added]
