# Supplementary figures and images for: Antithrombotic Effects of Combined PAR (Protease-Activated Receptor)-4 Antagonism and Factor Xa Inhibition
Source: Arterioscler Thromb Vasc Biol. 2020 Sep 10;40(11):2678–85. doi: 10.1161/ATVBAHA.120.314960 (PMC7571844; doi:10.1161/ATVBAHA.120.314960)

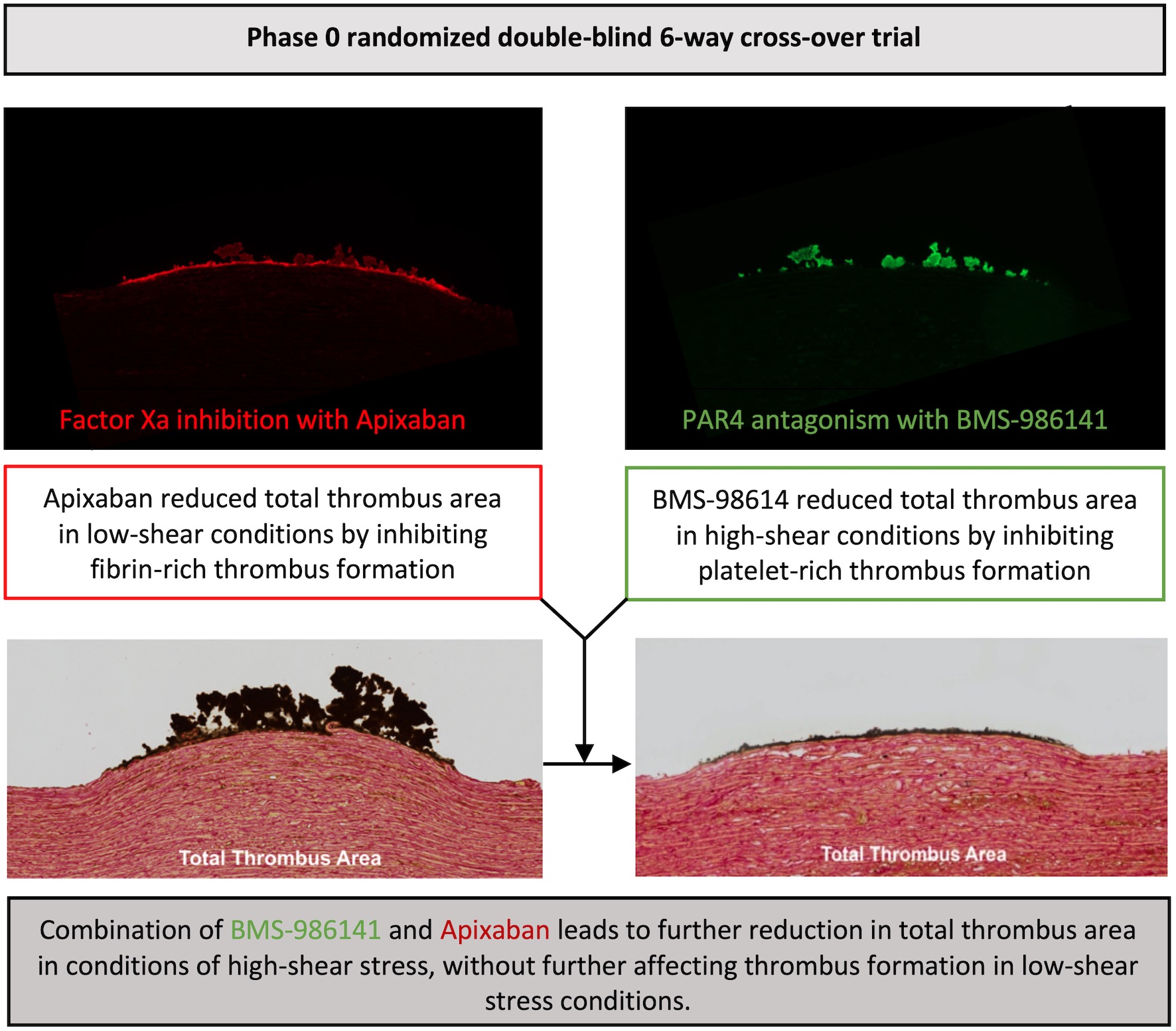

Supplement: Supplementary file 2 [file atv-40-2678-s002.jpg]
